# Plant cyanogenic glycosides: from structure to properties and potential applications

**DOI:** 10.3389/fpls.2025.1612132

**Published:** 2025-07-31

**Authors:** Beáta Piršelová, Jana Jakubčinová

**Affiliations:** Department of Botany and Genetics, Faculty of Natural Sciences and Informatics, Constantine the Philosopher University in Nitra, Nitra, Slovakia

**Keywords:** cyanogenic glycosides, structure and biosynthesis, biological function, content and distribution, toxicity, anticancer potential

## Abstract

Cyanogenic glycosides (CGs) represent an important group of secondary metabolites predominantly of plant origin, characterized by their ability to release hydrogen cyanide upon enzymatic hydrolysis. These compounds are widely distributed across the plant kingdom, where they play a crucial role in defense against herbivores and pathogens. In recent years, advanced analytical tools have greatly expanded our knowledge of CGs by enabling the identification of less abundant forms. Based on the latest data from published scientific studies, this review presents a comprehensive overview of CGs, with a focus on their structural variability, biosynthetic pathways, ecological functions, and inherent toxicity. Special attention is given to the quantity and distribution of significant CGs in plants, as the available data is often heterogeneous, fragmented, and dispersed across the literature. Furthermore, the review explores emerging evidence regarding the biomedical relevance of selected CGs, including their putative anticancer properties and broader therapeutic potential. The findings presented in this review may be applied in fields such as pharmacology, toxicology, food safety, and plant biotechnology - either to enhance CG content for crop protection or, conversely, to eliminate such content in order to improve food safety.

## 1 Introduction

**Figure 1:**
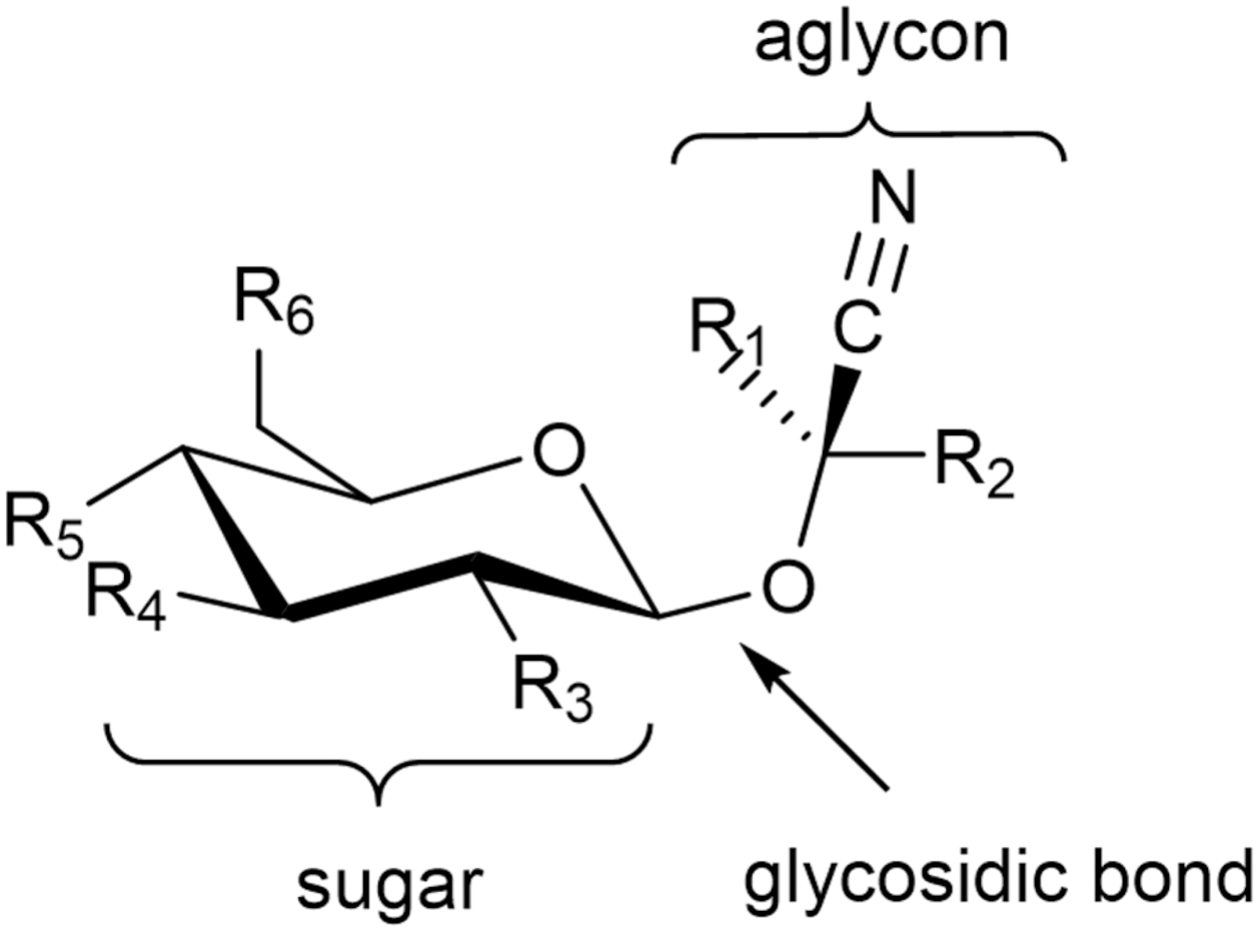
General structure of cyanogenic glycosides. R1 represents a proton for amygdalin, prunasin, and dhurrin and a methyl group for linamarin, while R2 is a variable organic group. R3–R6 represent variable inorganic (most commonly the hydroxyl group) or organic groups.

**Figure 2:**
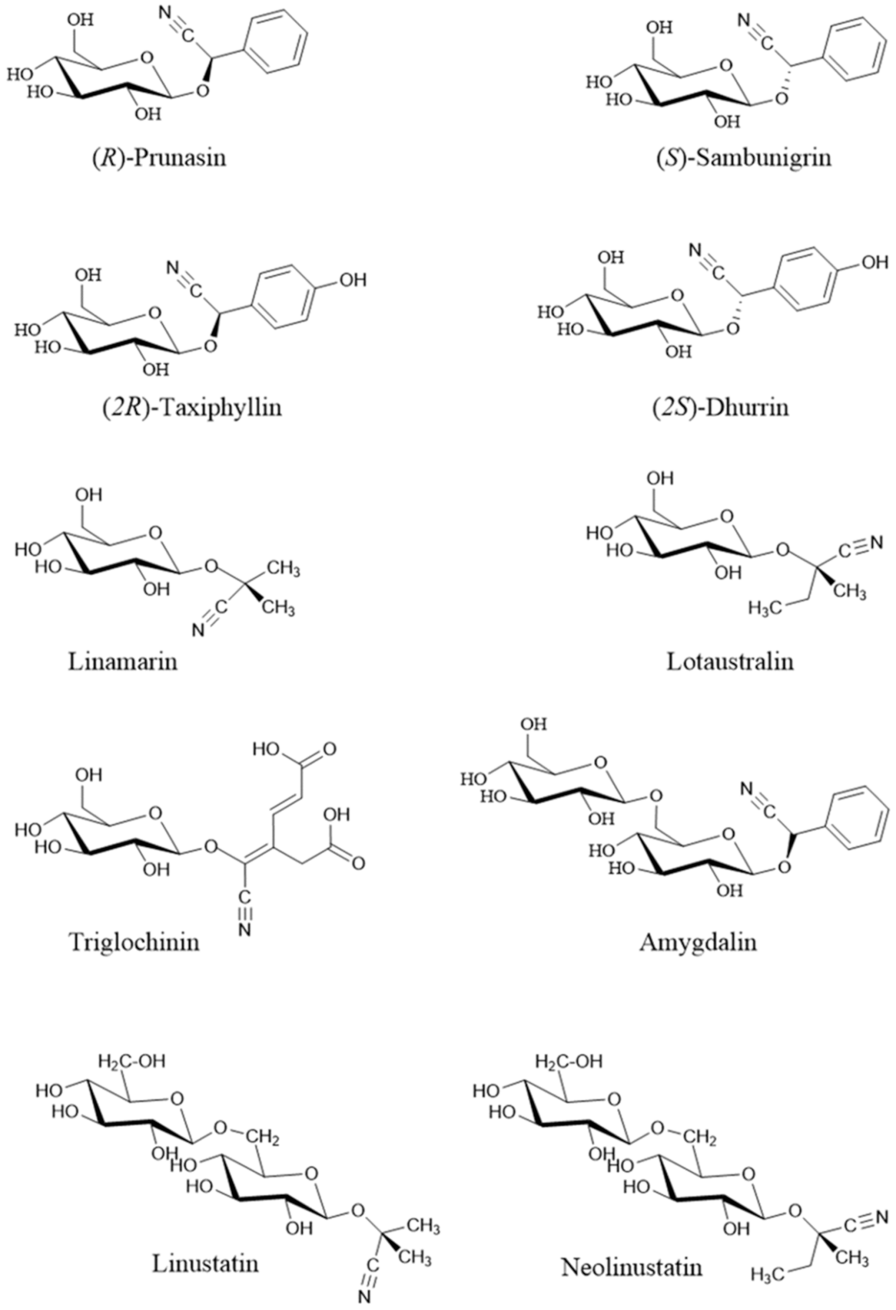
Structure of the most important cyanogenic glycosides (Rietjens and Eisenbrand, 2022). Stereoisomers: prunasin (*R*) / sambunigrin (*S*) and (*2R*)-taxyphyllin / (*2S*)-dhurrin.

Cyanogenic glycosides (cyanoglycosides, CGs) are secondary metabolites of predominantly plant origin and account for nearly 90% of the broader group of plant toxins known as cyanogens (Nampoothiri, 2017). Chemically, CGs are α-hydroxynitrile glucoside consisting of two main components: a sugar moiety - most commonly glucose - and an aglycone, the non-sugar part of the molecule that contains the cyanogenic group (CN). These components are linked through a glycosidic bond. Glycosylation plays a crucial role in determining the stability, solubility, and biological activity of CGs, including their potential antitumor properties (Mosayyebi et al., 2020). It also influences the interaction between the aglycone and cellular structures, such as receptors and proteins, thereby affecting a compound’s biological function (Pelley, 2012). The aglycone can vary in its chemical structure, most commonly appearing as aliphatic, cyclic, aromatic, or heterocyclic compounds. This part of the molecule largely determines the toxicity of CGs. Natural cyanogenic glycosides display considerable structural diversity in both their sugar and aglycone components (Vetter, 2017). Some naturally occurring CGs exist as stereoisomers, for example: (*R*)-lotaustralin/(*S*)-epilotaustralin, (*R*)- prunasin/(*S*)- sambunigrin, and (*2R*)-taxyphyllin/(*2S*)-dhurrin (Yulvianti and Zidorn, 2021). The general structure of CGs is illustrated in **Figure 1**, with the structures of the most significant compounds shown in **Figure 2**. The chemical diversity of plant CGs are described in more detail in article Yulvianti and Zidorn (2021).

Trivial names of CGs are usually derived from the Latin names of the plants from which they were first isolated (e.g. almond amygdalin - *Prunus amygdalus*). However, several isolated CGs do not have trivial names.

**Figure 3:**
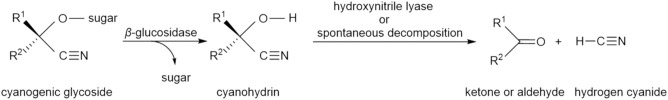
Glycoside cleavage – cyanogenesis.

Currently, 112 distinct CGs are known from the plant kingdom (Yulvianti and Zidorn, 2021). For plants they are important as protection against being consumed by animals and also as protection against various microorganisms (Zagrobelny et al., 2018). But actually, this protection is not provided by CG itself, but rather by the toxic hydrogen cyanide (HCN) released from stored CGs, cyanolipids, or cyanohydrins (Lechtenberg, 2011). This process occurs in an acidic environment (at low pH) or under the influence of hydrolytic enzymes with the formation of free HCN after the mechanical disruption of tissues. Cyanogenesis occurs in two phases: Phase 1 - cleavage of the carbohydrate component, Phase 2 - cleavage of the aglycone to aldehyde or ketone and HCN (**Figure 3**).

While CGs are stored in vacuoles, β-glucosidases are localized in the apoplastic space, bound to cell walls in dicotyledonous plants, and in the cytoplasm and chloroplasts in monocotyledonous plants. Hydroxynitrile enzymes accumulate mainly in the cytoplasm and plasma membranes. When plant tissue is disrupted, CGs and enzymes come into contact, and the CGs degrade into cyanohydrins, HCN, and ketones. The different compartmentalization of CGs and enzymes helps prevent excessive HCN production and its toxicity in plants (Vetter, 2017). Yet the cause of the typical bitter odor in the mechanical disruption of seeds containing CGs is not HCN, but the released benzaldehyde (Griffin, 1974; Moertel et al., 1982). CGs are also a re-mobilizable reservoir of reduced nitrogen, and increase plant tolerance by reducing oxidative stress and may support seedling development (Sanchez-Perez et al., 2009; Pičmanová et al., 2015). Moreover, free cyanide, including that released from the CGs, may act as a signaling molecule (Siegień and Bogatek, 2006).

## 2 Biosynthesis of plant cyanogenic glycosides

**Figure 4:**
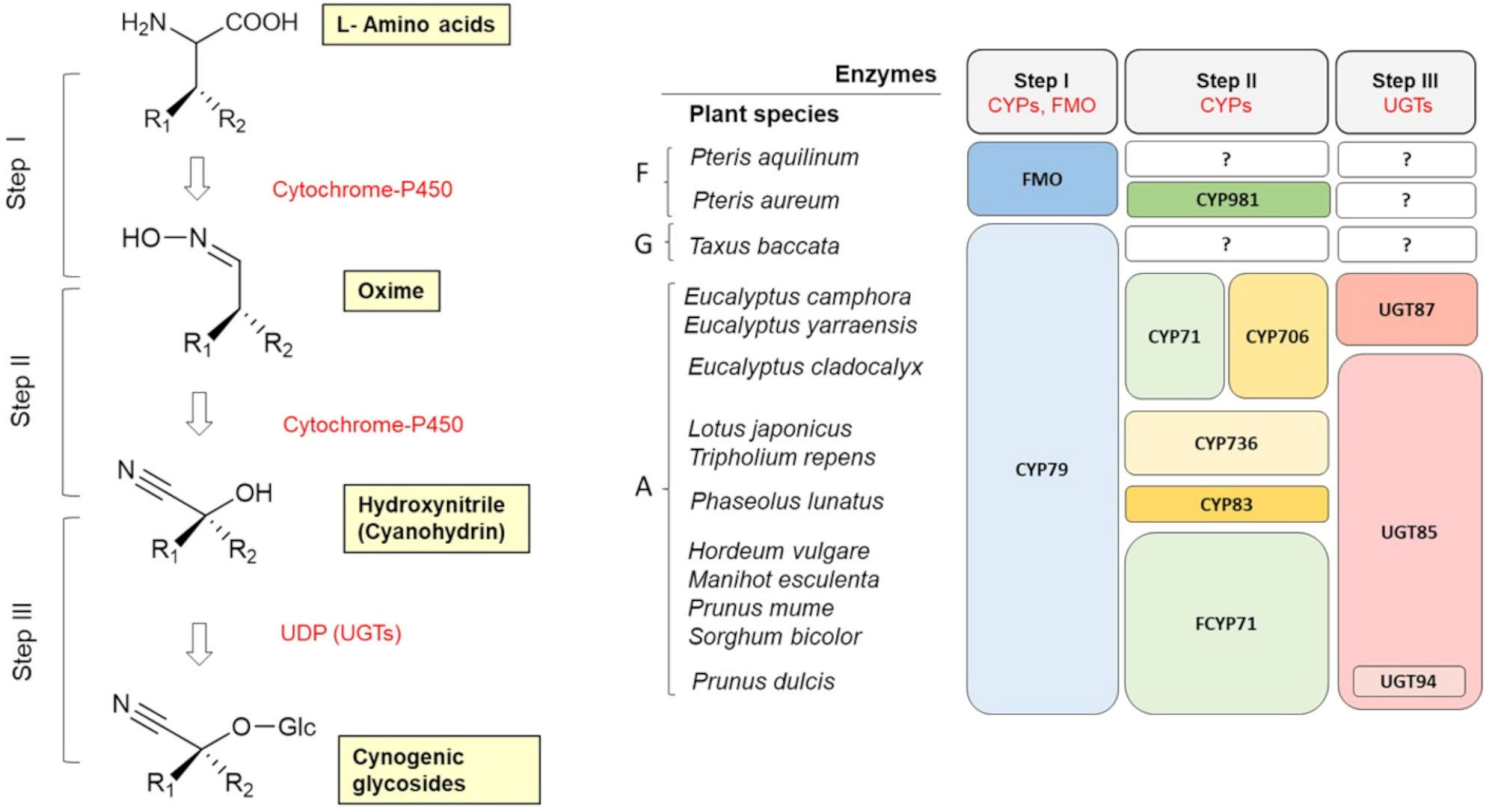
General scheme of the biosynthesis of cyanogenic glycosides in plants (adapted from Ganjewala et al., 2010). Evolution of key enzymes in cyanogenic glycoside biosynthesis in ferns (F), gymnosperms (G) and angiosperms (A) (adapted from Sánchez-Pérez and Neilson, 2024). CYP, cytochrome P450; FMO, flavin-containing monooxygenase; UGT, UDP-glucosyltransferase; ‘?’ denotes an unknown step.

Cyanogenic glycosides (CGs) are primarily derived from aliphatic amino acids (L-valine, L-isoleucine, L-leucine) and aromatic amino acids (L-phenylalanine, L-tyrosine). However, certain CGs - such as deidaclin, gynocardin, acalyphin, cycasin, and ranunculin - are synthesized from non-proteinogenic precursors (Nyirenda, 2020). While cyanogenic ferns and gymnosperm species predominantly produce aromatic CGs, angiosperms are known to synthesize both aliphatic and aromatic forms (Bak et al., 2006). To date, amino acid-derived cyanogenic glucoside pathways have been elucidated in various plant species. Despite species-specific variations, three conserved enzymatic steps have been identified across all CG biosynthetic pathways (**Figure 4**): 1. *Amino acid hydroxylation* – the conversion of α-amino acids to aldoximes via N-hydroxylated derivatives, mediated by membrane-bound enzymes from the cytochrome P450 (CYP) family. In gymnosperms and angiosperms, this is functionally conserved as the enzyme CYP79. 2. *Cyanohydrin formation* – the transformation of aldoximes into unstable cyanohydrins via further P450 cytochrome enzymes. In angiosperms, several more or less specific CYPs involved in this pathway have been characterized (CYP71, CYP706, CYP736). 3. *Glycosylation -* the attachment of a glucose unit, which stabilizes the cyanohydrins into cyanogenic glucosides. This step is catalyzed by the enzyme UDP-glucosyltransferase (in angiosperms, UGT85 and UGT94 have been characterized).

Transcription factors of the basic helix-loop-helix (bHLH) type play a key role in the regulation of CGs biosynthesis (Harun and Mohamed-Hussein, 2024). The plasticity of CYP gene expression, combined with their catalytic versatility, has made them key drivers of evolutionary innovation in plant secondary metabolism, allowing plants to colonize new environments and co-evolve with herbivores and pathogens (Bak et al., 2006; Xu et al., 2015).

In recent decades, significant progress has been made in the study of CG biosynthetic pathways and their regulation, which has enabled a deeper understanding of plant adaptation mechanisms and their evolutionary processes. This topic has been explored in more detail in studies by Forslund et al. (2004); Bak et al. (2006); Morant et al. (2007); Sun et al. (2018); Thodberg et al. (2020); Yulvianti and Zidorn (2021); Boter and Diaz (2023); Harun and Mohamed-Hussein (2024).

## 3 Genetic and ecological aspects of cyanogenesis

Cyanogenesis was first described in white clover (*Trifolium repens*) (Mirande, 1912), and it soon became evident that this species is polymorphic in terms of cyanogenesis – that is, both cyanogenic and acyanogenic plants occur within the same population (Armstrong et al., 1913). It was shown that this form of ecological adaptation results from polymorphism (the presence or absence) of genes responsible for both the synthesis of CGs (Ac) and the synthesis of β-glucosidases, enzymes that break down CGs (Li) (Hughes, 1991). Plants that carry at least one dominant (functional) allele at both genes (Ac and Li) are cyanogenic, while the occurrence of two nonfunctional alleles (ac and li) at either gene confers the acyanogenic phenotype. The Ac gene corresponds to the gene encoding cytochrome P450 from the CYP79D protein subgroup (specifically CYP79D15). CYP79D orthologs catalyze the first step in the biosynthesis of cyanogenic glycosides (**Figure 4**) (Olsen et al., 2008, 2013).

This chemical defense polymorphism is among the most long-studied and best-documented examples of adaptive polymorphism in plants. More cyanogenic plants are found in warmer and more humid regions with higher herbivore activity. However, since cyanogenesis is quite energetically costly, cyanogenic plants exhibit slower growth and reproduction in these areas. This represents a classic example of an evolutionary trade-off between defense and growth. It should, however, be noted that not all cyanogenic plants exhibit adaptive polymorphism. In many species, cyanogenesis is genetically fixed - either all individuals are cyanogenic, or none are. Adaptive polymorphism, as thoroughly documented in *Trifolium repens*, represents a specific evolutionary phenomenon that occurs in only certain species where selective pressures maintain both the presence and absence of cyanogenic expression within the same population (Olsen et al., 2008).

## 4 Distribution and content of cyanogenic glycosides in plants

CG synthesis is relatively widespread in the plant kingdom. More than 3000 plant species belonging to 130 families are cyanogenic (Yadav et al., 2023), including ferns, gymnosperms and angiosperms. In the agricultural context, the main sources of CGs are seeds and by-products of crops such as flax (*Linus usitatissimum*), apricot (*Prunus armeniaca*), bitter almond (*Prunus dulcis*), sorghum (*Sorghum vulgare*), wheat (*Triticum aestivum*), barley (*Hordeum vulgare*), oat (*Avena sativa*), cassava (*Manihot esculenta*) and apple (*Malus pumila*) (Hegnauer, 1986; Jones, 1998). In general, CGs exhibit a highly specialized distribution, with a given type of CG typically occurring in only one or two plant families. Furthermore, individual plant species generally produce only one or two types of CGs, reflecting their metabolic specialization and ecological adaptations (Bolarinwa et al., 2014a; Süli et al., 2017). Amygdalin and prunasin, for example, are predominantly found in plants of the *Rosaceae* family (e.g. *Prunus* spp., *Malus* spp.), where it functions as a chemical defense against herbivores. Linamarin and lotaustralin are characteristic of tropical and subtropical plants from the *Fabaceae* and *Euphorbiaceae* families (e.g., *Phaseolus lunatus, Manihot esculenta*), primarily serving to protect these plants from insect herbivores and microbial pathogens. A representative cyanogenic glycoside of the *Poaceae* family is dhurrin, which is especially abundant in young leaves of *Sorghum bicolor*, where it enhances the plant’s resistance to herbivores during early developmental stages.

However, the defensive potential of CGs is also manifested in the process of plant adaptation to various abiotic stressors, such as drought, excessive moisture, mineral imbalance, frost, trampling, and herbicide exposure (Bolarinwa et al., 2014a). Moreover, the degree of HCN induction appears to differ depending on whether the stress is chronic or acute (Wheeler et al., 1990; Woodrow et al., 2002). In stressed plants, where photosynthetic rate is reduced, CGs may also provide a ready source of nitrogen, remobilized when the stress is alleviated (O’Donnell et al., 2013; Schmidt et al., 2018). Under stress conditions, they also reduce oxidative stress and regulate the transport of carbon and nitrogen in plants (Conn, 1980; Rosati et al., 2019). Younger plants contain CGs much more than older ones (Dreyer et al., 1981). Some plants are not completely cyanogenic, others are not cyanogenic throughout the growing season. Cereal leaves are cyanogenic for example, but the grains are not. Papaya and mango leaves are also cyanogenic, but the fruits are not. Drought, frost, and the use of nitrates and herbicides can increase their amount and thus their toxicity to animals (Busk and Møller, 2002). Seasonal changes in the cyanide content of some species have also been reported (Robakowski et al., 2016; Bartnik and Facey, 2017). The amount of the most significant CGs in plants (expressed as the equivalent amount of HCN) is given in **Table 1**. The absolute amounts of individual CGs are listed in **Tables 2**–**9**. However, the reported CG levels in plant tissues also depend on the method of extraction and determination, as well as on the genotype, plant age, soil condition, fertilizer application, climatic conditions, and other factors (Bolarinwa et al., 2014a; Tahir et al., 2024).

**Table 1:** Amount of cyanogenic glycosides in plants (mg HCN equivalents·kg-1 plant material).

Plant	The main cyanogenic glycoside in tissues	Total cyanogenic glycoside content, (mg HCN equivalents·kg^-1^ plant material)	References
**Bamboo**	Taxiphyllin	shoots	
*Bambusa* spp.		1 000 – 8 000	Bhargava et al., 1996
*Bambusa* spp.		70 – 8 000	Feeley et al., 2012
*Bambusa balcooa*		1 150 – 2 420 (base – tip)	Sarangthem and Hoikhokim, 2010
*Bambusa balcooa*		620 – 2 150 (base – tip)	Choudhury et al., 2010
*Bambusa balcooa*		883 – 3 177 (base – apex)	Hoikhokim and Sarangthem, 2016
*Bambusa balcooa*		1 108	Rawat et al., 2015
*Bambusa bambos*		678	Rawat et al., 2015
*Bambusa khasiana*		2 180 – 2 877 (base – apex)	Hoikhokim and Sarangthem, 2016
*Bambusa tulda*		280 – 170 (base – tip)	Choudhury et al., 2010
*Bambusa tulda*		1 400	Sarma, 2018
*Bambusa tulda*		1 412	Rawat et al., 2015
*Bambusa pallida*		130 – 270	Choudhury et al., 2010
*Bambusa pallida*		1 180 – 2 232 (base – apex)	Hoikhokim and Sarangthem, 2016
*Bambusa pallida*		210	Sarma, 2018
*Bambusa vulgaris*		512	Chaturvedi et al., 2023
*Bambusa arundinacea*		1 010 – 1 060	Haque and Bradbury, 2002
*Bambusa auriculata*		150	Sarma, 2018
*Dendrocalamus* spp.		515 – 1 951	Rawat et al., 2015
*Dendrocalamus asp. Back.*		140	Pattarathitiwat et al., 2021
*Dendrocalamus giganteus*		70	Sarma, 2018
*Dendrocalamus hamiltonii*		1 553 – 2 917 (base – apex)	Hoikhokim and Sarangthem, 2016
*Dendrocalamus hamiltonii*		1 620 – 2 150 (base – tip)	Sarangthem and Hoikhokim, 2010
*Dendrocalamus hamiltonii*		150 – 2 420 (base – tip)	Chaturvedi et al., 2023
*Dendrocalamus hamiltonii*		140	Sarma, 2018
*Dendrocalamus strictus*		2 047 – 2 147 (base – apex)	Hoikhokim and Sarangthem, 2016
*Dendrocalamus sikkimensis*		1 883 – 2 553 (base – apex)	Hoikhokim and Sarangthem, 2016
*Dendrocalamus hookeri*		1 003 – 1 917 (base – apex)	Hoikhokim and Sarangthem, 2016
*Chimonobambusa callosa*		27 – 40	Hoikhokim and Sarangthem, 2016
*Chimonobambusa callosa*		32	Rawat et al., 2015
*Thyrsostachys oliveri*		1 098	Rawat et al., 2015
*Thyrsostachys oliveri*		180 – 373	Hoikhokim and Sarangthem, 2016
*Thyrsostachys oliveri*		7 – 72	Hoikhokim and Sarangthem, 2014
*Ochlandra wightii*		220 – 283	Hoikhokim and Sarangthem, 2016
*Schizostachyum dullooa*		160 – 443	Hoikhokim and Sarangthem, 2016
*Cephalostachyum latifolium*		140 – 1 020	Hoikhokim and Sarangthem, 2016
*Pseudostachyum polymorphum*		110 – 287	Hoikhokim and Sarangthem, 2016
*Melocanna baccifera*		1 250 – 1 977	Hoikhokim and Sarangthem, 2016
*Melocanna baccifera*		285	Rawat et al., 2015
*Melocanna bambusoides*		350 – 1 810 (base – tip)	Choudhury et al., 2010
*Phyllostachys mannii*		36	Rawat et al., 2015
**Flax**	Linamarin	seeds	
*Linum* spp.	Linustatin	15 – 2 428	Waszkowiak et al., 2015
*Linum* sp.	Neolinustatin	2.5 – 3.9	Park et al., 2024
*Linum usitatissimum*	Lotaustralin	360 – 390	Haque and Bradbury, 2002
***Sorghum***	Dhurrin		
*Sorghum vulgare*	Amygdalin	750 – 790 leaves	Haque and Bradbury, 2002
*Sorghum* sp.		10 – 240	Aikman et al., 1996
*Sorghum halepense*		5 – 690	Giantin et al., 2024
*S. bicolor* × *S. sudanense*		83 – 1 235	Giantin et al., 2024
*Sorghum* sp.		122 310	Bolarinwa et al., 2016
*Sorghum* sp.		0.06 seeds	Park et al., 2024
**Almond**	Amygdalin		
*Prunus amygdalus*, bitter		300 – 4 700	Feeley et al., 2012
*Prunus amygdalus*, bitter		918 – 1 215	Chaouali et al., 2013
*Prunus amygdalus*, sweet		16.2 – 32.4	Chaouali et al., 2013
**Peach**	Amygdalin		
*Prunus persica*	Prunasin	710 – 720 kernels	Haque and Bradbury, 2002
*Prunus* sp.	Dhurin	0.192 powder	Park et al., 2024
**Plum**	Amygdalin		
*Prunus* sp.		696 – 764 kernels	Haque and Bradbury, 2002
**Nectarine**	Amygdalin		
*Prunus persica* var. *nucipersica*		196 – 209 kernels	Haque and Bradbury, 2002
**Apricot**	Amygdalin		
*Prunus armeniaca*	Prunasin	785 – 813 stone	Haque and Bradbury, 2002
*Prunus armeniaca*	Taxiphyllin	0.064 fruit	Park et al., 2024
*Prunus armeniaca*	Dhurrin	0.502 seeds	Park et al., 2024
*Apricot* sp.		540 – 1 193.4 kernels	Chaouali et al., 2013
**Apple**	Amygdalin		
*Malus* spp.	Prunasin	690 – 790 seeds	Haque and Bradbury, 2002
*Malus* spp.	Sambunigrin	0.17 seeds	Park et al., 2024
**Giant taro**	Triglochinin		
*Alocasia macrorrhizos*		29 – 32 leaves	Haque and Bradbury, 2002
**Cherry**	Prunasin		
*Prunus* spp.		0.03 seeds	Park et al., 2024
**Loquat**	Prunasin		
*Eriobotrya japonica*	Taxiphyllin	0.75 seeds	Park et al., 2024
**Lima beans**	Lotaustralin		
*Phaseolus lunatus*	Linamarin	7.59	Park et al., 2024
*Phaseolus lunatus*	Lotaustralin	10 – 400	Shlichta et al., 2014
**Quince**			
**Cydonia oblonga**	Prunasin	0.03 seeds	Park et al., 2024
**Elderberry**	Sambunigrin		
*Sambucus nigra*, black elderberry	Prunasin	1 033.22 leaves	Senica et al., 2019
*Sambucus nigra*	Amygdalin	414.23 flowers	Senica et al., 2019
*Sambucus nigra*		54.88 berries	Senica et al., 2019
*Sambucus ebulus*, dwarf elderberry		8.76 leaves	Senica et al., 2019
*Sambucus ebulus*		58.19 flowers	Senica et al., 2019
*Sambucus ebulus*		26.25 berries	Senica et al., 2019
*Sambucus racemose*, red elderberry		1.05 leaves	Senica et al., 2019
*Sambucus racemose*		4.45 flowers	Senica et al., 2019
*Sambucus racemose*		3.12 berries	Senica et al., 2019
**Cocoyam**	Amygdalin		
*Colocasia esculenta*, purple		10 840	Bolarinwa et al., 2016
*Colocasia esculenta*, white		6 290	Bolarinwa et al., 2016
*Colocasia esculenta*, cream		5 880	Bolarinwa et al., 2016
*Colocasia esculenta*		740 tubers	Igbadul et al., 2014
*Colocasia esculenta*		21	Abdulrashid and Agwunobi, 2009
*Colocasia esculenta*		17	Olajide et al., 2011

**Table 2:** Amygdalin content in plants.

Source	Amygdalin content (mg·kg^-1^)	Reference
*Prunus serotina*	20 – 950 leaves	Santos Pimenta et al., 2014
*Prunus serotina*	2 – 680 seeds	Bolarinwa et al., 2014b
*Prunus avium*	3 – 890 seeds	Bolarinwa et al., 2014b
*Prunus amygdalus*	120 fruit	Bolarinwa et al., 2014b
*Prunus amygdalus*	370 – 1 458	Yildirim et al., 2014
*Prunus amygdalus*, bitter	40 060	Lee et al., 2013
*Prunus amygdalus*, light bitter	992	Lee et al., 2013
*Prunus amygdalus*, sweet	63	Lee et al., 2013
*Prunus armeniaca*	14 – 370 seeds	Bolarinwa et al., 2014a
*Prunus armeniaca*	13 – 500 kernels	Haque and Bradbury, 2002
*Prunus armeniaca*	8 610	Yildirim and Askin, 2010
*Prunus mume*	17 – 490 seeds	Bolarinwa et al., 2014b
*Prunus domestica*	440 – 17 490 seeds	Bolarinwa et al., 2014b
*Prunus domestica*	12 – 700 kernels	Haque and Bradbury, 2002
*Prunus persica*	6 – 810 seeds	Bolarinwa et al., 2014b
*Prunus avium*	3 – 890 red fruit	Bolarinwa et al., 2014b
*Prunus persica* var. *nucipersica*	120 seeds	Bolarinwa et al., 2014b
*Malus domestica*	950 – 3 910 seeds	Bolarinwa et al., 2015
*Malus domestica*	690 seeds	Jaszcak-Wilke et al., 2021
*Manihot esculenta*, cassava	8 840 – 48 330 seeds	Bolarinwa et al., 2016
*Sambucus nigra*	190 leaves	Senica et al., 2019
*Sambucus nigra*	22.82 flowers	Senica et al., 2019
*Sambucus nigra*	4.91 berries	Senica et al., 2019
*Sambucus ebulus*	5.88 leaves	Senica et al., 2019
*Sambucus ebulus*	40.97 flowers	Senica et al., 2019
*Sambucus ebulus*	18.95 berries	Senica et al., 2019
*Sambucus racemose*	0.36 leaves	Senica et al., 2019
*Sambucus racemose*	2.68 flowers	Senica et al., 2019
*Sambucus racemose*	0.68 berries	Senica et al., 2019
*Eriobotrya japonica*	5 900 seeds	Tanaka et al., 2020

**Table 3:** Prunasin content in different plant species/foods.

Source	Prunasin content (*mg·L^-1^ or mg·kg^-1^)	Reference
*Eriobotrya japonica*	8.77 seeds, powder	Park et al., 2024
*Eriobotrya japonica*	8.14 seeds	Park et al., 2024
*Malus* spp.	0.85 – 1.83 seeds	Park et al., 2024
*Prunus avium*	0.308 seeds	Park et al., 2024
*Prunus persica*	2.059 powder seeds	Park et al., 2024
*Prunus persica*	2.614 – 2.911 pulp (canned)	Park et al., 2024
*Prunus persica*	3.663 – 4.435 canned form	Park et al., 2024
*Prunus persica*	110 roots	Mfarrej and Sharaf, 2011
*Prunus persica*	95 leaves	Mfarrej and Sharaf, 2011
*Prunus amygalus*	644 roots	Mfarrej and Sharaf, 2011
*Prunus amygalus*	509 leaves	Mfarrej and Sharaf, 2011
*Prunus dulcis*	2 – 750 roots	Mfarrej and Sharaf, 2011
*Prunus dulcis*	575 leaves	Mfarrej and Sharaf, 2011
*Prunus armeniaca*	5.41 seeds	Park et al., 2024
*Prunus armeniaca*	0.66 fruit	Park et al., 2024
*Prunus armeniaca*	0.93 seeds	Park et al., 2024
*Prunus armeniaca*	230 roots	Mfarrej and Sharaf, 2011
*Prunus armeniaca*	212 leaves	Mfarrej and Sharaf, 2011
*Prunus domestica*	253 roots	Mfarrej and Sharaf, 2011
*Prunus domestica*	190 leaves	Mfarrej and Sharaf, 2011
*Prunus mume*	2.52 beverages	Park et al., 2024
*Prunus mume*	0.32	Park et al., 2024
*Prunus mume*	1.20 – 1.40 axis	Park et al., 2024
*Prunus mume*	2.95* juice	Park et al., 2024
*Prunus mume*	0.13* fruit syrup	Park et al., 2024
*Prunus mume*	0.03* vinegar	Park et al., 2024
*Prunus laurocerasus*	35 – 110 kernels	Demirbolat and Kartal, 2018
*Prunus laurocerasus*	max. 900 pulp	Demirbolat and Kartal, 2018
*Prunus laurocerasus*	12 500 – 16 500 leaves	Demirbolat and Kartal, 2018
*Sambucus* sp.	0.154 beverages	Park et al., 2024
*Sambucus nigra*	26.27 leaves	Senica et al., 2019
*Sambucus nigra*	12.13 flowers	Senica et al., 2019
*Sambucus nigra*	27.48 berries	Senica et al., 2019
*Sambucus ebulus*	2.40 leaves	Senica et al., 2019
*Sambucus ebulus*	16.84 flowers	Senica et al., 2019
*Sambucus ebulus*	6.78 berries	Senica et al., 2019
*Sambucus racemose*	0.37 leaves	Senica et al., 2019
*Sambucus racemose*	1.13 flowers	Senica et al., 2019
*Sambucus racemose*	0.920 berries	Senica et al., 2019

**Table 4:** Linamarin content in different plant species.

Source	Linamarin content (mg·kg^-1^)	Reference
*Linum* spp.	20 – 140	Russo and Reggiani, 2014
*Linum* spp.	11.88 seeds	Roulard et al., 2017
*Prunus amygdalus*	251 – 901 raw	Amjadian et al., 2020
*Manihot esculenta*	190.65 – 921.13 roots	Zhong et al., 2020

**Table 5:** Lotaustralin content in different plant species.

Source	Lotaustralin content (*μg·L^-1^ or μg·kg^-1^)	Reference
*Linum* sp.	12 600 seeds	Roulard et al., 2017
*Linum* sp.	24 034 – 37 734 seeds	Park et al., 2024
*Linum* sp.	793.86* oil	Park et al., 2024
*Linum* sp.	41 134 – 45 067 sprouted	Park et al., 2024
*Linum* sp.	4 157 – 6 627 roasted	Park et al., 2024
*Linum* sp.	16 067 stemmed	Park et al., 2024
*Phaseolus lunatus*	73 – 434 bean*s*	Park et al., 2024
*Manihot* sp.	770 000 – 1 040 000 leaves	Chongtham et al., 2022
*Manihot* sp.	25 000 – 27 000 roots	Chongtham et al., 2022
*Manihot* sp.	307 starch powder	Park et al., 2024
*Manihot* sp.	2 640 – 3 034 starch pearl	Park et al., 2024
*Rhodiola rosea*	8 060 000	Wang and Ruan, 2005
*Rhodiola rosea*	1 060 000 – 1 350 000 roots	Gryszczyńska et al., 2013
*Rhodiola kirilowii*	53 773 – 74 791 roots	Gryszczyńska et al., 2013

**Table 6:** Sambunigrin content in elderberries.

Source	Sambunigrin content (μg·kg^-1^)	Reference
*Sambucus nigra*	80 – 770	Pascariu and Israel-Roming, 2022
*Sambucus nigra*	18 800	Senica et al., 2016
*Sambucus nigra*	1 006 750 leaves	Senica et al., 2019
*Sambucus nigra*	379 290 flowers	Senica et al., 2019
*Sambucus nigra*	22 490 berries	Senica et al., 2019
*Sambucus ebulus*	480 leaves	Senica et al., 2019
*Sambucus ebulus*	380 flowers	Senica et al., 2019
*Sambucus ebulus*	620 berries	Senica et al., 2019
*Sambucus racemose*	320 leaves	Senica et al., 2019
*Sambucus racemose*	640 flowers	Senica et al., 2019
*Sambucus racemose*	1 520 berries	Senica et al., 2019

**Table 7:** Dhurrin content in different plant species.

Source	Dhurrin content (*μg·L^-1^ or μg·kg^-1^)	Reference
*Sorghum* sp.	840 000 – 7 140 000 stems	Zhong et al., 2020
*Sorghum* sp.	1 630 000 – 6 570 000 roots	Zhong et al., 2020
*Sorghum halepense*	104 – 10 717	Giantin et al., 2024
*Sorghum halepense*	57 000 – 7 961 000	Giantin et al., 2024
*S. bicolor* x *S. sudanense*	957 – 10 717 000	Giantin et al., 2024
*Prunus persica*	43.87 seeds	Park et al., 2024
*Diospyros* sp., (persimmon)	76.22* juices	Park et al., 2024
*Prunus mume*	58.81* wine	Park et al., 2024
*Prunus armeniaca*	84.13 seeds	Park et al., 2024
*Prunus armeniaca*	43.19	Park et al., 2024
*Manihot* sp.	108.50 chips	Park et al., 2024
*Manihot* sp.	78.71* pressed juice	Park et al., 2024

**Table 8:** Linustatin and neolinustatin content in flax.

Source	Cyanogen glycoside	Linustatin and neolinustatin content (mg·kg^-1^)	Reference
*Linum* spp.	Linustatin	220 – 2 830 seeds	Zhao et al., 2019
	Linustatin	24 – 910 seeds	Roulard et al., 2017
	Neolinustatin	1 760	Zhao et al., 2019
	Neolinustatin	38 – 460 seeds	Roulard et al., 2017
	Neolinustatin	280 – 950	Russo and Reggiani, 2014
	Linustatin	300 – 850	Russo and Reggiani, 2014

**Table 9:** Taxiphyllin content in different plant species.

Source	Taxiphyllin content (*μg·L^-1^ or μg·kg^-1^)	Reference
*Bambusa* sp.	266 000 – 434 000 fresh, unprocessed shoots	Sang-A-Gad et al., 2011
	248 000 – 299 000 fresh sliced shoots	Sang-A-Gad et al., 2011
	39 000 – 196 000 sliced pickled shoots left over for 1 night	Sang-A-Gad et al., 2011
	22.36 – 53.80 canned shoots	Park et al., 2024
*Eriobotrya japonica*	27.25 seeds	Park et al., 2024
*Eriobotrya japonica*	68.9 seed powder	Park et al., 2024
*Prunus mume*	129.68 dried	Park et al., 2024
*Prunus mume*	65.6 – 87.03 axis	Park et al., 2024
*Prunus mume*	34.45* vinegar	Park et al., 2024
*Prunus* sp.	134.49* fruit syrup	Park et al., 2024

Some specialized herbivores (mainly insects) preferentially feed on cyanogenic plants and use them as protection against predators. Several arthropod species (e.g., *Diplopoda*, *Chilopoda*, *Insecta*) can even synthesize CGs *de novo*. The unique plant-insect interaction based on CG is extensively discussed in the study by Zagrobelny et al. (2018).

## 5 Technologies for reducing cyanogenic glycoside content in foods

CGs are considered antinutrients that reduce the quality of feed and food, causing various health issues in animals, including humans. It is recommended that such plants be treated prior to consumption to minimize HCN content. Different types of processing methods are used to reduce CG content in plants. The most important processing methods include drying, grinding, dipping, peeling, ultrasound-assisted detoxification, autoclaving, soaking, boiling and fermentation (Bolarinwa et al., 2014a). The latter has proven to be highly effective, for example, in reducing CG content in bamboo shoots (Chongtham et al., 2022). In the process of acid fermentation of certain CGs, the bacteria *Lactobacillus plantarum*, *Bacillus subtilis*, *Bacillus licheniformis*, and *Bacillus sonorensis* proved to be effective (Abban et al., 2013; Menon et al., 2015). Sun drying after retting reduces cyanide content by 98.6%. Boiling/cooking can reduce free cyanide content by 96% within 15 minutes. After heating for 25 minutes, bound cyanide is reduced by 55% (Nampoothiri, 2017; Chongtham et al., 2022). A reduction of cyanides by 93% was also achieved by applying sodium bicarbonate (5 mL of a 0.4% NaHCO_3_ solution) to 1 g of cassava leaves (Latif et al., 2019). Conserved stone fruit must be peeled because cyanides also occur in the resulting infusion up to 33 mg·kg^-1^ HCN. However, the processing methods applied are not always sufficiently effective, and a certain amount of CG remains in plant products, thus posing potential health risks. **Tables 2**-**9** also show varying amounts of CG in differently processed products. The issue of reducing cyanide content in plants and processed products is further explored by Rawat et al. (2015); Bolarinwa et al. (2016); Tahir et al. (2024) and others. Studies have also been developed to estimate the risks associated with the daily intake of CGs in food (Schrenk et al., 2019; Park et al., 2024).

## 6 Cyanogenesis, cyanide detoxification in plants and animals

When assessing the harmful effects of substances involved in cyanogenesis, the focus is mostly on the effects of released HCN; other components (intact glycosides and their hydrolysis products) do not appear to be serious in terms of acute toxicity. HCN is extremely toxic to animals, including humans. The lethal HCN dosage in most animal species is in the range of 2 mg·kg^-1^ to 2.5 mg·kg^-1^, with the exception of pandas (Clarke et al., 1981; Panter, 2018). The acute oral lethal dose of HCN for humans is reported to be 0.5 – 3.5 mg·kg^-1^ of body weight (Halstrom and Moiler, 1945). The permissible limit of cyanogen content in food is 500 mg·kg^-1^ (Food and Agriculture Organization, 2005).

HCN toxicity in animals, including humans, is due to blocking the release of energy from ATP (adenosine triphosphate) by inhibiting cytochrome oxidase activity in the respiratory chain (**Figure 4**). Hence the tissues and cells of the organisms are unable to utilize the oxygen that is transported by the blood, which can lead to internal suffocation (Gracia and Shepherd, 2004). The most important laboratory finding in cyanide poisoning is metabolic acidosis with dramatically increased lactate concentration (Baud et al., 2002) (**Figure 4**). The effects of HCN on the ability of the thyroid gland to store and process iodine are also documented (Erdogan, 2003). Clinical signs of acute poisoning include rapid breathing, decreased blood pressure and rapid pulse, dizziness, convulsions, vomiting, and blue discoloration of the skin due to lack of oxygen. As cellular hypoxia worsens, consciousness progresses to coma. Symptoms appear within seconds to minutes.

Cyanide detoxification in plants and animals is a critical biochemical process that helps mitigate the toxic effects of cyanogenic compounds. Both plants and animals have evolved mechanisms to detoxify or tolerate cyanide to survive in environments where these compounds are prevalent. The primary mechanism for cyanide detoxification in most plants is the β-cyanoalanine pathway. In this process, cyanide reacts with the amino acid L-cysteine to form β-cyanoalanine, catalyzed by the enzyme β-cyanoalanine synthase (CAS). This reaction occurs mainly in the mitochondria and is the major route by which plants detoxify endogenous cyanide. β-cyanoalanine can be further converted into asparagine, aspartate, and ammonia by β-cyanoalanine hydratase or nitrilase, integrating the cyanide-derived nitrogen into the plant’s nitrogen metabolism (Velišek and Hajšlova, 2009). The β-cyanoalanine synthase pathway is described in more detail by Machingura et al. (2016).

**Figure 5:**
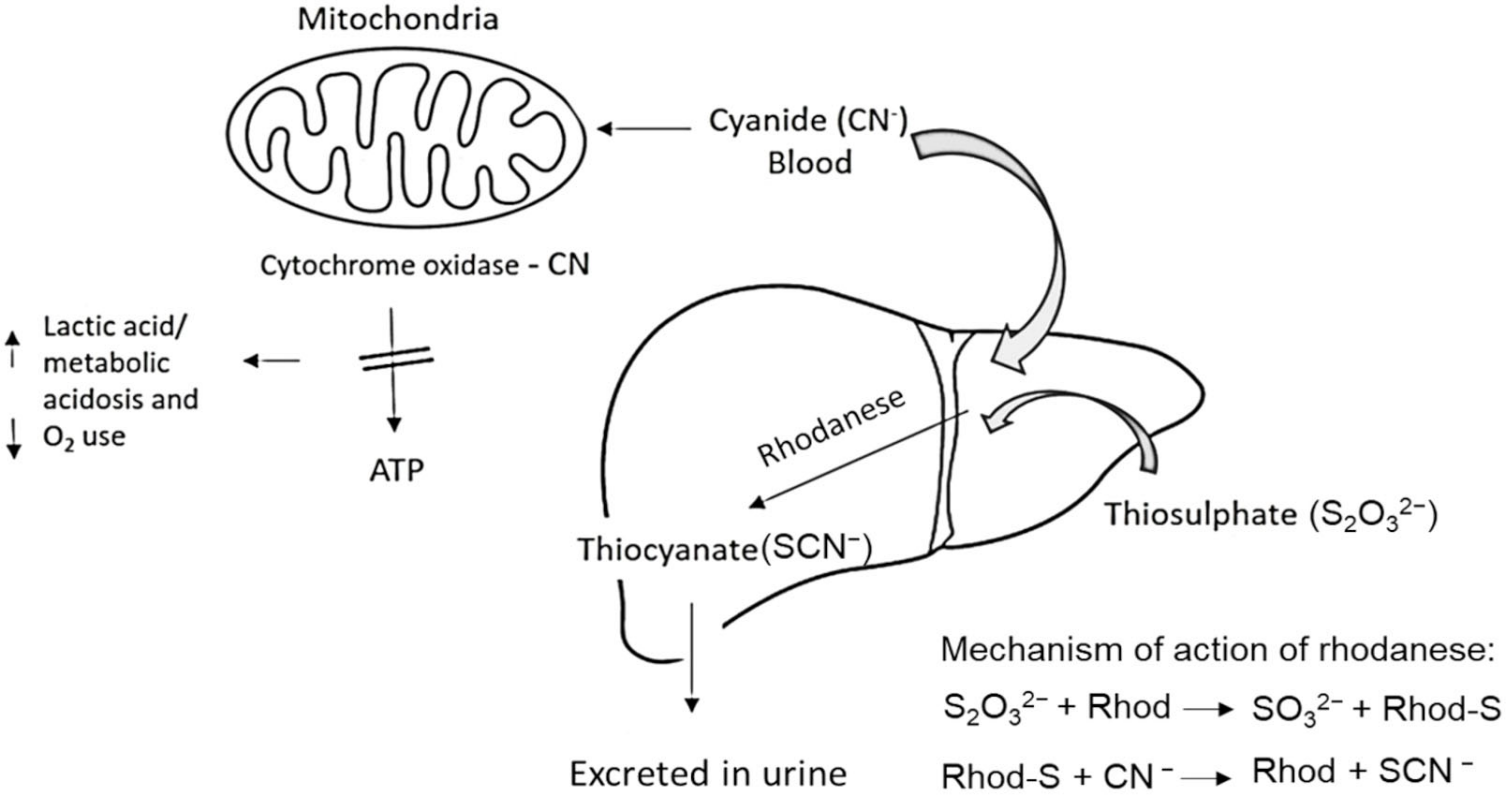
Detoxification of cyanide in animals. Cyanide, primarily absorbed through the skin and gastrointestinal tract, is relatively quickly converted in the liver by the enzyme rhodanese (Rhod) into the less toxic thiocyanate, which is excreted in the urine. Excess cyanide binds to cytochrome oxidase in mitochondria, leading to the inhibition of ATP production and the cessation of aerobic metabolism. The result is systemic hypoxia and the potentially death of the individual (adapted from: Gracia and Shepherd, 2004; Aussignargues et al., 2012).

The most significant detoxification system in animals is the rhodanese enzyme system, which converts cyanide into thiocyanate, which is much less toxic and can be safely excreted through the urine (Gracia and Shepherd, 2004) (**Figure 5**). A further manner of detoxification is the binding of cyanide to hydroxocobalamin (vitamin B12), resulting in the formation of nontoxic cyanocobalamin.

The ability of an animal to tolerate certain doses of HCN also depends on the animal species, body weight, digestion rate, type of food, and the animal’s ability to detoxify the released HCN. The lethal dose for sheep is 2.4, cattle 2.0, mice 3.7, cats 2.0, 0, rats 0.5 – 10.0 and dogs 1.5 mg·kg^-1^ body weight (Jones, 1998). Ruminants are more sensitive to HCN poisoning because the enzymes that facilitate the release of HCN are destroyed by gastric HCl in these animals. Of this group, goats appear to be the most susceptible to cyanide (Patel et al., 2014). The specifics of CG poisoning in ruminants are described in detail by Gensa (2019). In non-ruminants, CGs are partially cleaved, and HCN is released only by the action of the colonic microflora where the pH is more suitable for the action of glycosides. But the hydrolysis is not complete, some glycosides are absorbed in their original form. In ruminants, many bacteria found in the rumen can hydrolyze CGs, with the degree of effectiveness depending on glycoside type and feed ration. The composition of gut microbiota also plays a significant role in the tolerance of mammals to the content of secondary metabolites in their diet. The gut microbiome of the giant panda and red panda contains a higher proportion of *Pseudomonas bacteria* compared to other mammals. Their microbiome is thus enriched with genes that encode the enzymes involved in the potential degradation or detoxification of HCN (Zhu et al., 2018). This is likely an evolutionary adaptation, that is not unique in the context of the plant kingdom, and can also be observed in some animals or microorganisms (Panter, 2018). Lemurs and gorillas also possess the unique ability to utilize high cyanide content in their diet without any acute or chronic harmful effects (Ballhorn et al., 2016). The metabolism of CGs in animals is described in more detail in the article by Cressey and Reeve (2019). However, estimating the health risks related to consuming CG-containing plants is often not straightforward, as sometimes the entire plant, including seeds, is consumed, while in other cases only the fruit or other parts are eaten. However, chronic intoxication can occur if plants containing CGs are part of the daily diet and consumed in larger quantities (Hartanti and Cahiani, 2020). More detailed data on the daily intake of CGs in selected foods is documented by Park et al. (2024).

## 7 Diseases caused by the toxic effects of cyanoglycosides

Chronic cyanide toxicity causes several diseases, especially in tropical areas where the main food is plant-based. Growth retardation, goiter, and cretinism are relatively common diseases in developing countries where people consume food with very low iodine content (<100 μg/day) and high cyanide content (Odo et al., 2014; Bolarinwa et al., 2016). High and sustained intake of cyanogens in sublethal concentrations of manioc (cassava) flour in combination with the low intake of sulfur amino acids also causes konzo - a disease of the upper motor neurons characterized by irreversible but non-progressive symmetric spastic para/tetraparesis which mostly affects children and women in the tropics (Howlett et al., 1990). Konzo disease is common during periods of drought, or when there is a shortage of alternative foods due to unfavorable social or environmental factors (Nzwalo and Cliff, 2011). Practically identical to konzo disease is mantakassa (Howlett et al., 1990; Bruyn and Poser, 2003). Tropical ataxic neuropathy (TAN) is another health problem associated with the continual consumption of improperly processed cassava products, especially in Africa (e.g. Nigeria). TAN is used to describe several neurological syndromes attributed to toxicolutricative causes. Symptoms of TAN include tongue pain, optic atrophy, neurosensory deafness, and sensory gait ataxia (Bolarinwa et al., 2016). Diseases caused by CGs are described in more detail in the study by Nyirenda (2020).

## 8 Significant cyanogenic glycosides

### 8.1 Amygdalin

Amygdalin ([(6-*O*-β-D-glucopyranosyl-β-D-glucopyranosyl)oxy](phenyl)acetonitrile, D-mandelonitrile β-D-gentiobioside) was isolated from apricot stones [*Prunus dulcis* (Mill.) D. Webb var. amara (DC.) H. Moore] by Pierre-Jean Robiquet and Antoine François Boutron Charlard in 1830 (Rosen and Shorr, 1979). As one of the most common CGs, it occurs in 1,200+ plant species. Amygdalin is highly concentrated in plants of the *Rosaceae*, *Caprifoliaceae*, and *Oleaceae* families (Hösel, 1981). Amygdalin is colorless with a melting point of 213°C, insoluble in non-polar solvents, and is highly soluble in ethanol and moderately soluble in water. Its highest concentrations are found in the seeds of fruits, which have a characteristic bitter taste due to the presence of amygdalin. Apricot seeds contain the highest amount of amygdalin, up to 2 – 2.5% by weight in most varieties of apricots (**Table 2**). Amygdalin content is significantly lower in seedless fruits (0.01 – 2.96 mg·g^-1^) and also in processed products (0.004 – 0.12 mg·g^-1^). In commercially available apple juices, the amygdalin content ranges from 0 to 0.007 mg·ml^-1^ (Bolarinwa et al., 2014b). A semisynthetic derivative of amygdalin is the structurally different laetrile (mandelonitrile-β-glucuronide) (He et al., 2020).

Amygdalin’s effects began to be studied from the late 1960s until the mid-1980s, whereby such research included tests to determine the chronic and acute toxicity and teratogenicity of amygdalin. The results showed that this substance’s toxicity depends on the manner of administration and dose. Adverse effects were shown to the least extent when administered intravenously and intramuscularly, while higher toxicity was recorded when administered orally (Beamer et al., 1983). With oral amygdalin intake, HCN poisoning from plant sources is not manifested until after a certain latency period, namely approximately 15–60 minutes (Kolesárová et al., 2021). The lethal dose for humans of intravenous injection of amygdalin is 5 g (Qadir and Fatima, 2017). The consumption of 50 bitter almonds is deadly for adults. However, for young children, 5–10 almonds are fatal (World Health Organisation, 2012). Higher doses of amygdalin causes symptoms of intoxication, nausea, and bluing of the skin. Regular use of amygdalin can cause nervous system problems and, ultimately, its disabling. Intoxication in humans is manifested by headache, dizziness and confusion, in severe cases by paralysis, coma, and death of the affected person (Sadoff et al., 1978). Omelka et al. (2021) focused their study on the effect of amygdalin on human osteoblast functions *in vitro*, and demonstrated that amygdalin at high concentrations (10 mg·ml^-1^) negatively affects osteoblasts, increases bone resorption, and reduces osteoblast viability. At high concentrations it also had a negative impact on the oxidative balance of male reproductive structures (Ďuračka et al., 2016).

Yet amygdalin is considered an important component of alternative medicine due to its wide range of healing effects (Kolesárová et al., 2021). At lower doses, it has positive effects in the treatment of asthma, bronchitis, diabetes, leprosy, vascular lesions, and sickle cell disease (Fukuda et al., 2003; Makarević et al., 2014; Song and Xu, 2014; Zhou et al., 2020). It can also relieve fevers, coughs, and thirst. Traces of released HCN and benzaldehyde from the amygdalin molecule can eliminate the occurrence of bacteria in the oral cavity, which is the cause of tooth decay and bad breath (Griffin, 1974). However, the anticancer effects of amygdalin have attracted the most attention. The use of bitter almond derivatives in the treatment of skin tumors is mentioned in 5,000-year-old Egyptian papyri. In the 1920s, apricot kernels were recognized in many states as a preventive and malignant inhibitor of cancer cell growth. The success of apricot kernels in cancer treatment was also supported by the American biochemist Ernst Theodore Krebs, who was the first to present amygdalin under the incorrect designation “vitamin B17” (Krebs, 1970) and believed that together with diet and vitamins, this substance could prevent cancer growth (Chandler et al., 1984). Amygdalin administration became one of the most popular and unconventional anti-cancer treatments in the 1970s, and has been used by 70% of American cancer patients since 1978 (Barakat et al., 2022). Howard and Miller (1984) report that while laetrile has shown little antitumor activity in animal studies, no antitumor activity has been reported in clinical trials in human populations. A similar conclusion was reached by Jaszcak-Wilke et al. (2021). Critics of amygdalin use warn that amygdalin is ineffective and even toxic, and say that its accumulation leads to severe poisoning (Blaheta et al., 2016). Side-effects associated with laetrile toxicity reflect symptoms of cyanide poisoning, including liver damage, difficulty walking, fever, subsequently coma, and eventually death. Amygdalin is still marketed as an anti-carcinogenic “vitamin B17” in many countries, and the United States (where this theory originated) has long proven its danger and ineffectiveness as a cancer treatment (Süli et al., 2017). But the results of many molecular biology studies have again highlighted the increased anti-tumor potential of amygdalin (Fukuda et al., 2003; Makarević et al., 2014; Song and Xu, 2014; Zhou et al., 2020). In the case of colon cancer, a decrease in the expression of many genes associated with growth functions, apoptosis and trafficking was observed at amygdalin concentrations of 0.25–5 mg·ml^-1^ (Kim et al., 2016). When tested on other cell lines with a concentration of 10 mg·ml^-1^ of amygdalin, the growth rate of breast cancer cells (MCF-7 and MDA-MB-231) was inhibited (Lee and Moon, 2016), sometimes leading to a decrease in cell motility or a reduction in the ability to synthesize collagen and fibronectin in the case of kidney cancer cells (Caki 1, A498, KTC-26) (Luo et al., 2016). The physiological and therapeutic effects of amygdalin are described in more detail in Kolesárová et al. (2021).

### 8.2 Prunasin

The cyanogenic monoglycoside prunasin (Prulaurasin, Laurocerasin, (2*R*)-(β-D-glucopyranosyloxy)(phenyl)acetonitrile, D-Mandelonitrile β-D-glucoside) is formed by removing one of the two β-D-glucopyranosyl groups from amygdalin with the enzyme β-glucosidase (Ellenhorn and Barceloux, 1997). Prunasin is a component of over 3,000 plant species, occurring mainly in plant tissues of the families *Myrtaceae*, *Saxifragaceae*, and *Scrophulariaceae*, and especially found in the families *Rosaceae* and *Polypodiaceae* (Vetter, 2017). *Prunus (P.)* species containing prunasin include e.g. *P. armeniaca* (apricot), *P. dulcis* (bitter almond), *P. persica* (peach), *P. serotina* (black/wild cherry), *P. virginiana* (red almond) and *P. laurocerasus* (cherry laurel) (Hodgson, 2012; Demirbolat and Kartal, 2018) (**Table 3**). Prunasin is synthesized by plants to protect seeds during maturation. Evident proof of this is provided by the study by Demirbolat and Kartal (2018), which highlighted the increasing content of prunasin in seeds (from an initial 3.5 mg·100 g^-1^ to 11 mg·100 g^-1^). During fruit maturation, such content decreases and eventually disappears. In the leaves, the average content is maintained (1250 to 1650 mg·100 g^-1^). To date, there is little data on the toxicokinetics of prunasin in humans. Prunasin intoxication due to the frequent consumption of some plants is common especially in tropical areas, and is associated with motor neuron diseases, such as console and mantakassa (Howlett et al., 1990; Bruyn and Poser, 2003). Toxic effects are particularly evident when fruits or preparations containing prunasin are taken concomitantly with foods containing high levels of β-glucosidase enzymes (such as apple and pear seeds).

In experiments performed in 2003 under *in vivo* and *in vitro* conditions, the antitumor activity of this compound was observed. It comprised the strong inhibition of the activation of the Epstein-Barr virus antigen induced by the tumor promoter. The researchers also observed a delay in the onset of skin cancer in mice (Fukuda et al., 2003). Prunasin can also be found in extracts from *Prunus mume (Ume)*, which exhibit hepatoprotective, anti-inflammatory, antioxidant, antibacterial and anticancer properties. MK615 is a mixture of extracts containing Ume-derived hydrophobic substances (Morimoto-Yamashita et al., 2012). The antitumor properties of MK615, along with other extracts from *Prunus mume*, have been studied. Research has shown that MK615 inhibits proliferation and induces apoptotic cell death in a variety of cancer cells, including those from both solid and hematological tumors (Bailly, 2020). *P. amygdalus* var. *amara* treatment also significantly decreased cancer cell growth in most cancer cell lines, when doses and exposure time were taken into consideration (Shalayel et al., 2023).

### 8.3 Linamarin

Linamarin (formerly called phaseolunatin, 2-(β-D-glucopyranosyloxy)-2-methyl-propanenitrile, α-hydroxyisobutyronitrile β-D-glucose) is a derivative of valine and isoleucine. The data on linamarin content in plant tissues is very limited (**Table 4**). It is found in the leaves and roots of plants such as almond (*Prunus amygdalus*), flax (*Linum usitatissimum*) and manioc (*Manihot esculenta*). In manioc, linamarin represents more than 80% of all CGs (Kuete, 2014). Manioc, also known as cassava or yuca, is a major source of carbohydrates for some 500 million people worldwide, particularly in Africa, where it is the third most important food source. According to linamarin content, hot and sweet varieties of cassava are distinguished with both varieties being commonly consumed. But under certain circumstances, it becomes dangerous and even fatal for humans. Although cassava juice contains low protein content, it also contains a relatively large amount of CGs, especially linamarin and lotaustralin (Nassar and Dorea, 1982). These CGs are hydrolyzed in the presence of the enzyme linamarase (Hösel, 1981). Chronic linamarin poisoning is manifested by the occurrence of endemic tropical ataxic neuropathy (TAN), especially in the elderly, the development of console disease, and deteriorating health with a number of symptoms resulting from iodine deficiency (Howlett et al., 1990; Ernesto et al., 2002). Recent studies have highlighted the potential antineoplastic effect of linamarin, especially when administered with the activating enzyme linamarase. The application of linamarin together with linamarase shows cytotoxic effects against several cancer cell lines, including HT-29, MCF-7, Caov-3, and HeLa (Yusuf et al., 2006; Idibie et al., 2007; Mosayyebi et al., 2020). The toxicity of cyanide released during the action of linamarase is eliminated by using so-called ‘suicide gene therapy’, the principle of which involves introducing the desired gene into a cancer cell to convert non-toxic compounds into toxic substances at the tumor site (Zarogoulidis et al., 2013). The principle of this mixture’s cytotoxic effect is described in more detail by Liyanage et al. (2024). Song and Xu (2014) also mention the possible mechanism of linamarin, where the effect of HCN on the mitochondrial respiratory chain can lead to the death of cancer cells.

### 8.4 Lotaustralin

Lotaustralin (2-hydroxy-2-methylbutyronitrile-β-D-glucopyranoside or (2*R*)-2-(β-D-gluc opy- ranosyloxy)-2-methylbutanenitrile) is a CG found in plants of the families *Linaceae* (e.g. *Linum usitatissimum*), *Euphorbiaceae* (e.g. *Manihot esculenta*), *Fabaceae* (e.g. *Phaseolus lunatus*), and *Crassulaceae* (e.g. *Rhodiola rosea*) (Pulido and Gill, 2013) (**Table 5**). The methyl derivative of linamarin – lotaustralin and linamarin itself are the two main CG compounds in foods derived from manioc roots, while the content of HCN and cyanohydrins is generally low (Butter, 1965; Mlingi et al., 1995). The sweet type of cassava contains 50 times lower levels of this CG compared to its bitter version. The concentration of lotaustralin increases significantly, especially during the dry season (Bovell-Benjamin and Roberts, 2016). Compared to linamarin, as the main cyanogenic component (93%) present in manioc lotaustraline content is much lower (7%) (Liangcheng et al., 1995).

### 8.5 Sambunigrin

Sambunigrin (also known as L-prunasin or (2*S*)-(β-D-glucopyranosyloxy)-(phenyl)- acetonitrile) was isolated in 1905 from the leaves of black base (*Sambucus nigra*) by the French scientists, pharmacists and botanists J.L.L. Guignard and Dr. E. Bourquelot. It also occurs in the American species *S. racemosa - S. calicarpa* Greenea, *S. microbotrys* Rydberg (Hegnauer, 1989). Much less sambunigrin occurs in the North American elder (*Sambucus canadensis*) (Bohm and Glennie, 1971; Buhrmester et al., 2000). Sambunigrin content means that the unripe fruits of black elder must not be consumed directly. Likewise, all green parts of the plant contain toxic CGs. However, the flowers and ripe fruits no longer contain this substance. The (*R*) diastereomer of sambunigrin is *R*-prunasin. Heat treatment causes the decomposition of sambunigrin into compounds that are harmless to the human body (Młynarczyk et al., 2018).

In addition to sambunigrin, other CGs, such as amygdalin, dhurrin, prunasin, linamarin, zierin, and holocalin, have also been detected in the tissues of various elderberry species (Knudsen and Kaack, 2015; Appenteng et al., 2021). The most abundant include sambunigrin, amygdalin and prunasin (Senica et al., 2016, 2019; Pascariu and Israel-Roming, 2022), where sambunigrin predominates in red elderberry, prunasin in black elderberry, and amygdalin in dwarf elderberry (Senica et al., 2019).

### 8.6 Dhurrin

Dhurrin ((2*S*)-(β-D-glucopyranosyloxy)(4-hydroxyphenyl)acetonitrile, (*S*)-4-hydroxy- mandelonitrile β-D-glucoside) is a CG produced in many plants elonging to *Poaceae*, *Rosaceae*, *Araliaceae*, *Proteaceae*, *Betulaceae*, *Chenopodiaceae*, *Proteaceae*, *Boraginaceae* etc (Miller et al., 2006; Yadav et al., 2023). Dhurrin, discovered in several varieties of Sorghum in 1906 as being responsible for bovine poisoning by HCN, is most often associated with the species *Sorghum bicolor* (*Poaceae*) (Mao and Anderson, 1965). Although dhurrin provides plants with an effective defense against most herbivores; however, some beetles and aphids have developed mechanisms that allow them to resist its toxic effects (Pentzold et al., 2014). Dhurrin occurs in whole plants except mature seeds (grains) (Yadav et al., 2023), and it is toxic mainly to farm animals that consume it (Kojima et al., 1979). The biosynthesis, catabolism, and toxicity of dhurrin are described in more detail in Yadav et al. (2023). The concentration of dhurrin in tissues decreases with the age of plants, and is highest during seed germination when it reaches about 30% of shoots’ dry matter. Its content in tissues also increases due to various stress factors, mainly drought (Busk and Møller, 2002; Emendack et al., 2018). In addition, high concentrations of nitrate, also potentially toxic to ruminants, may accumulate during or shortly after periods of drought (Yadav et al., 2023). Sorghum malt contains a high amount of dhurrin (up to 1.400 mg·kg^-1^) that is insufficiently degraded in the malting process, so African beers may contain higher amounts of cyanide (about 11 mg·kg^-1^) (Tokpohozin et al., 2016). Dhurrin content in different plant species is shown in **Table 7**.

### 8.7 Linustatin and neolinustatin

Linustatin (2-{[6-*O*-(β-D-glucopyranosyl)-β-D-glucopyranosyl]oxy}-2-methylpropanenitrile) and neolinustanin [(2*R*)-2-{[6-*O*-(β-D-glucopyranosyl)-β-D-glucopyranosyl]oxy}-2-methyl-butane-nitrile)] are soluble in water and forms a weakly acidic solution. These CGs have so far been quantified only in flax (**Table 8**). Until recently, linamarin had been considered the main glycoside in flax seeds; however, much higher levels of the diglycosides linustatin and neolinustatin have been demonstrated (Russo and Reggiani, 2014). Therefore, consuming flax seeds in large quantities is not recommended. According to Daun et al. (2003), to reach acute cyanide toxicity, a person would need to consume eight cups (1 kg) of ground flaxseed. With the recommended daily intake of about one to two tablespoons, approximately 5–10 mg of HCN would be released (Rosling, 1994). When consuming ground flaxseed, the bioavailability of HCN and human exposure levels are higher than when consuming whole flaxseeds or when they are heat-treated. Cassava contains significantly more CGs than flaxseed (Touré and Xueming, 2010). Some sources (Smith et al., 1980) suggest that these CGs can protect rats from the toxic effects of selenium.

### 8.8 Taxiphyllin

Taxiphyllin is the (R)-enantiomer of dhurrin (2*R*)-(β-D-glucopyranosyloxy)(4-hydroxyphenyl)acetonitrile or (*R*)-4-hydroxymandelonitrile β-D-glucoside). Taxiphyllin is a CG found in bamboo shoots, *Sorghum bicolor* and *Henriettella fascicularis* (Calderón et al., 2003). Taxiphyllin is highly unstable and thermolabile. Although the taxiphyllin content in bamboo shoots is much higher than in cassava roots, the cyanide content in bamboo shoots decreases substantially following harvesting and processing. An approximately 80% reduction in CGs was achieved after vacuum freeze-drying for 24 hours at −50°C (Rawat et al., 2015). Taxiphyllin content in bamboo shoots is shown in **Tables 1**, **9**. As mentioned earlier, pandas can withstand the toxic effects of bamboo CGs due to the composition of their gut microbiome. Taxiphyllin content in different plant species is shown in **Table 9**.

### 8.9 Triglochinin

Triglochinin ((2*Z*,4*E*)-4-[cyano(β-D-glucopyranosyloxy)methylene]-2-hexenedioic acid) was isolated from the flowers of the monocotyledonous plant *Triglochin maritimum* L (Eyjólfsson, 1970). Using chromatographic methods, two isomers of this compound were identified. This tyrosine-derived CG was later found in the tissues of *Alocasia macrorrhiza*, *Thalictrum aquilegiifolium*, and some plants from the *Araliaceae* family (*Aralia* sp*inosa*) (Lechtenberg et al., 2022), as well as the *Arecaceae* family (Nahrstedt, 1975). The content of triglochinin in tissues is limited to certain periods of collection or developmental stages. In the case of *A.* sp*inosa*, the flower buds collected in July showed the highest content of triglochinin, just below 0.2% dry weight. There is very little data on triglochinin content in plant tissues. Chongtham et al. (2022) measured 29–32 mg·kg^-1^ (as HCN equivalents) of this CG in the tissues of giant taro (*Alocasia macrorrhizos*).

## 9 Detection of cyanogenic glycosides

The detection of CGs in food is important for public health protection, as improper food processing can release toxic cyanide that is highly harmful to humans. Additionally, the detection of these substances plays an important role in complying with food regulations, which set maximum allowable concentrations of CGs in various foods (Cressey and Reeve, 2019; Vetter, 2000). Many countries have already introduced regulations to reduce the risk of cyanide exposure from consuming foods that contain these compounds.

The detection of CGs depends on several factors (Cressey and Reeve, 2019; Tahir et al., 2024), such as:

1. *Type and concentration of CGs* – different types of CGs may have varying abilities to release cyanide at different concentration.
2. *Molecular structure of CGs* – differences in chemical structure affect how these glycosides behave during detection, and what methods are most suitable for their extraction and identification.
3. *Composition of an individual’s gut microbiome* – microbial content in the digestive system can influence the metabolism of CGs and cyanide production.
4. *Extraction method* – the way CGs are extracted from the sample (e.g., using different solvents, temperatures, or extraction times) affects the efficiency and accuracy of detection (Vetter, 2000).
5. *Quantification method used* – various analytical techniques (e.g., HPLC – High-Performance Liquid Chromatography, GLC = Gas-Liquid Chromatography) may have different sensitivities and specificities when measuring CGs content.
6. *Presence of other substances* – other compounds in the sample may interfere with the detection process or affect measurement accuracy.

**Figure 6:**
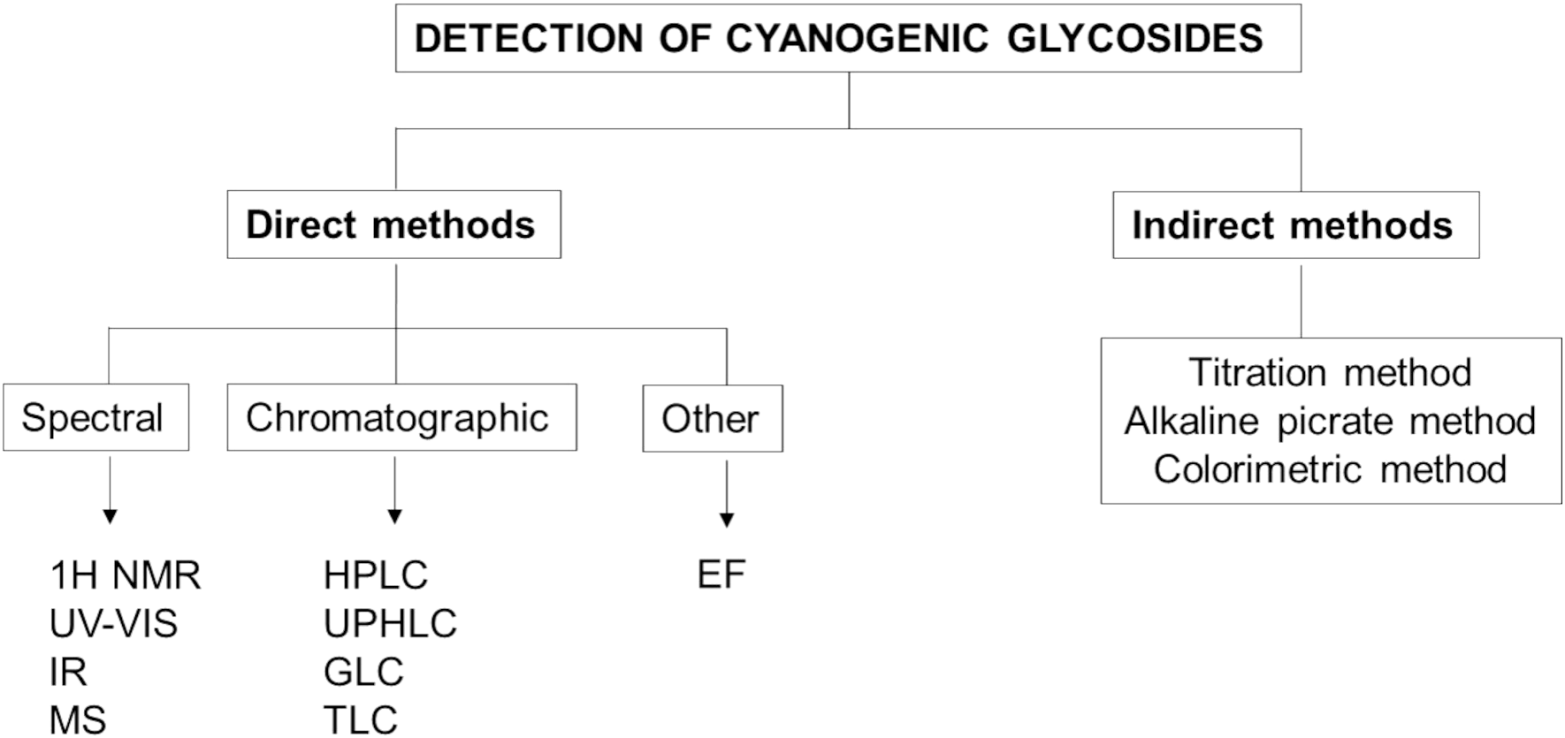
Methods used for the detection of cyanogenic glycosides (1H NMR, Proton Nuclear Magnetic Resonance; UV-Vis, Ultraviolet-Visible Spectroscopy; IR, Infrared Spectroscopy; MS, Mass Spectroscopy; HPLC, High-Performance Liquid Chromatography; UPHLC, Ultra-Performance Liquid Chromatography; GLC, Gas-Liquid Chromatography; TLC, Thin Layer Chromatography; EF, Electrophoresis).

CGs are quantified using direct and indirect methods of determination. The direct method targets CGs as the molecules of interest, while the indirect method focuses on the released HCN after hydrolysis (Azmi, 2019; Hartanti and Cahiani, 2020) (**Figure 6**). One of the most well-known indirect methods of determination is the Guignard sodium picrate test (Hartanti and Cahiani, 2020).

Many reviews summarize this issue. Analytical methods for the determination of amygdalin are clearly presented by Popa et al. (2021) who highlight various analytical methods with detailed parameters. Zhao et al. (2024) focus on the issue in the comparison of HPLC/UPLC methods for the determination of CGs. Risk assessment of food safety associated with foods containing CGs was addressed by Cressey et al., with a focus on rural New Zealand (Cressey et al., 2022).

## 10 Conclusion

Cyanogenic glycosides represent a broad group of structurally differing compounds with various biochemical properties. Some organisms use cyanogenic acids as protection against predators. These compounds are also present in many plants, which in some countries form an important part of the diet for local populations. The harmful effects of CGs on the human body are fairly well researched, and there is a vast database of scientific studies on their toxic properties. The risks associated with the consumption of processed and unprocessed plant parts containing these substances can now be more accurately estimated. Although cyanide itself is extremely toxic and can cause severe poisoning, some plants containing CGs are the subject of intensive research, especially for their potential in therapeutic applications. Current studies are focusing on the synthesis of derivatives of these compounds that have enhanced anti-tumor effects, which opens up new opportunities for cancer treatment. However, it is essential that the risks associated with the release of cyanide, which remains highly toxic, are not overlooked in this research. As a result, much research is focused on developing technologies and methods that allow the breakdown of cyanide compounds to be controlled or minimized with the aim to avoid adverse health effects.
